# Rivoceranib, a VEGFR-2 inhibitor, monotherapy in previously treated patients with advanced or metastatic gastric or gastroesophageal junction cancer (ANGEL study): an international, randomized, placebo-controlled, phase 3 trial

**DOI:** 10.1007/s10120-023-01455-5

**Published:** 2024-01-28

**Authors:** Yoon-Koo Kang, Min-Hee Ryu, Maria Di Bartolomeo, Ian Chau, Harry Yoon, Jong Gwang Kim, Keun-Wook Lee, Sang Chul Oh, Atsuo Takashima, Anna Kryzhanivska, Yee Chao, Ludovic Evesque, Michael Schenker, Arlo McGinn, Yufan Zhao, Jennifer Lee, Lucjan Wyrwicz, Narikazu Boku

**Affiliations:** 1 Department of Oncology, Asan Medical Center, University of Ulsan College of Medicine, 88, Olympic-Ro 43-Gil, Songpa-Gu, Seoul, 05505 Korea; 2 Fondazione IRCCS-Istituto Nazionale Tumori, Milan, Italy; 3 Royal Marsden Hospital, Sutton, UK; 4 Mayo Clinical Cancer Center, Rochester, MN USA; 5 Kyungpook National University Chilgok Hospital, Daegu, Gyeonggi-Do Korea; 6 Seoul National University College of Medicine, Seoul National University Bundang Hospital, Songnam, Korea; 7 Korea University Guro Hospital, Seoul, Korea; 8 National Cancer Center Hospital, Tokyo, Japan; 9 Ivano-Frankivsk Regional Oncological Center, Ivano-Frankivsk, Ukraine; 10 Taipei Veterans General Hospital, Taipei, Taiwan; 11 Centre Antoine-Lacassagne, Nice, France; 12 Centrul de Oncologie ‘Sf. Nectarie’, Sectia de Oncologie Medicala, Craiova, Romania; 13 Elevar Therapeutics, Inc, Fort Lee, NJ USA; 14 Klinika Onkologii I Radioterapii, Centrum Onkologii, Instytut Im.Marii Sklodowskiej-Curie, Warsaw, Poland

**Keywords:** Stomach neoplasms, Tyrosine protein kinase inhibitors, Vascular endothelial growth factor receptor-2

## Abstract

**Background:**

Rivoceranib is an oral, selective tyrosine kinase inhibitor of vascular endothelial growth factor receptor-2. ANGEL (NCT03042611) was a global, randomized, double-blinded, placebo-controlled, phase 3 study evaluating rivoceranib as 3rd-line or ≥4th-line therapy in patients with advanced/metastatic gastric or gastroesophageal junction (GEJ) cancer.

**Methods:**

Patients had failed ≥2 lines of chemotherapy and were randomized 2:1 to rivoceranib 700 mg once daily or placebo with best supportive care. Primary endpoint: overall survival (OS) in the intention-to-treat population. Secondary endpoints: progression-free survival (PFS), objective response rate (ORR), and disease control rate (DCR) by blinded independent central review (BICR).

**Results:**

In total, 460 patients (rivoceranib *n* = 308, placebo *n* = 152) were enrolled. OS was not statistically different for rivoceranib versus placebo (median 5.78 vs. 5.13 months; hazard ratio [HR] 0.93, 95% CI 0.74–1.15; *p* = 0.4724). PFS by BICR (median 2.83 vs. 1.77 months; HR 0.58, 95% CI 0.47–0.71; *p* < 0.0001), ORR (6.5% vs. 1.3%; *p* = 0.0119), and DCR (40.3 vs. 13.2%; *p* < 0.0001) were improved with rivoceranib versus placebo. In patients receiving ≥4th-line therapy, OS (median 6.34 vs. 4.73 months; *p* = 0.0192) and PFS by BICR (median 3.52 vs. 1.71 months; *p* < 0.0001) were improved with rivoceranib versus placebo. The most common grade ≥ 3 treatment-emergent adverse events with rivoceranib were hypertension (17.9%), anemia (10.4%), aspartate aminotransferase increased (9.4%), asthenia (8.5%), and proteinuria (7.5%).

**Conclusions:**

This study did not meet its primary OS endpoint. Compared to placebo, rivoceranib improved PFS, ORR, and DCR. Rivoceranib also improved OS in a prespecified patient subgroup receiving ≥4th-line therapy.

**Supplementary Information:**

The online version contains supplementary material available at 10.1007/s10120-023-01455-5.

## Introduction

Although gastric cancer is declining in incidence, it is still the fifth most common type of cancer worldwide in terms of incidence, with over 1 million new cases in 2020, and globally it is the fourth most common cause of cancer death after lung cancer, colorectal cancer, and liver cancer [1]. The incidence of gastric cancer and associated mortality vary substantially by region and are significantly related to diet and *Helicobacter pylori* infection [1–3]. Incidence rates are high in Eastern Asia and Latin America and low in Western developed countries [2]. In 2012, 60% of all newly diagnosed gastric cancer cases occurred in China, Japan, and Korea [2]. Age-standardized incidence rates for gastric cancer in these countries remain among the highest in the world at 22.7, 29.9, and 41.8 per 100,000 in China, Japan, and Korea, respectively [2]. The incidence of esophageal adenocarcinoma is increasing in the West, particularly in North America and Western Europe [4].

Clinical practice guidelines for the management of advanced/metastatic gastric cancer recommend doublet or triplet platinum/fluoropyrimidine combinations as 1st-line treatment for fit patients (doublet with trastuzumab for human epidermal growth factor receptor 2 [HER2]-positive patients) [5, 6]. In the 2nd-line setting, a taxane, irinotecan, or the anti-vascular endothelial growth factor receptor-2 (VEGFR-2) monoclonal antibody ramucirumab as single agent or ramucirumab in combination with paclitaxel for patients with performance status (PS) 0–1, is recommended. At the time of protocol finalization (June 13, 2016) there were no globally approved 3rd-line treatment regimens, although apatinib, a different name for rivoceranib, was approved in China in 2014 for use in this setting. However, before the end of the study, there were three new additions to approved standards of care for ≥3rd-line treatment in patients with advanced gastric cancer. The European Society for Medical Oncology (ESMO) guidelines were updated to recommend trifluridine/tipiracil for patients beyond 2nd-line treatment [7], pembrolizumab was approved in the USA for programmed death-ligand 1 (PDL1)-positive gastric cancer after progression on or after two or more lines of chemotherapy, and nivolumab was approved in East Asia for patients receiving ≥3rd-line therapy [8, 9]. Nevertheless, despite recent advances in treatment, the prognosis for advanced gastric cancer is generally poor, with a global 5-year survival rate of 5–10% [10].

Rivoceranib, also known as YN968D1 (the mesylate salt) is an orally administered, small-molecule, tyrosine kinase inhibitor (TKI) that selectively binds to and strongly inhibits VEGFR-2, reducing VEGF-mediated endothelial cell migration, proliferation, and tumor microvascular density [11]. Rivoceranib is the International Nonproprietary Names (INN)/generic name established in 2018. It was previously known as apatinib. Rivoceranib has also been shown to augment T-cell-mediated anti-tumor cytotoxicity [12]. In a phase 2 study [13] and a phase 3 study [14] conducted in China, apatinib significantly improved median overall survival (OS) and median progression-free survival (PFS) in patients with chemotherapy-refractory advanced/metastatic gastric carcinoma compared with placebo. The pharmacokinetics of rivoceranib have been shown to be similar in healthy male White, Japanese, and Chinese subjects, with no clinically or statistically significant differences observed in a phase 1 study [15]. In a phase 2 study of rivoceranib in multiple tumor types conducted in South Korea and USA, rivoceranib at a dose of 685 mg (850 mg as rivoceranib mesylate salt) once daily was well tolerated and showed promising efficacy in patients with gastric cancer; in the 15 patients with gastric cancer, there was one partial response (6.7%) and a disease control rate of 86.7%, with a median PFS of 6.93 months [16]. Based on these promising results, the global phase 3 ANGEL study was conducted to investigate the efficacy and safety of rivoceranib plus best supportive care (BSC) globally in White as well as in Asian (Taiwan, Japanese, and Korean) patients with advanced/metastatic gastric cancer.

## Methods

### Study design and patients

The ANGEL study (ClinicalTrials.gov registration number: NCT03042611) was a prospective, randomized, double-blinded, placebo-controlled, multicenter, multinational, phase 3 study to evaluate the efficacy and safety of rivoceranib plus BSC versus placebo plus BSC. Patients were enrolled at 95 sites in 12 countries, including the USA, Europe, and Asia.

Eligible patients were 18 years or older with a documented primary diagnosis of histologically or cytologically confirmed adenocarcinoma of the stomach or gastroesophageal junction, locally advanced unresectable, recurrent, or metastatic disease, and one or more measurable or non-measurable evaluable lesions according to Response Evaluation Criteria in Solid Tumors (RECIST) version 1.1 [17]. Other eligibility criteria included failure of or intolerance to at least two prior lines of standard chemotherapy with each line including one or more of the following agents: fluoropyrimidine (intravenous [iv] 5-fluorouracil [5-FU], capecitabine, or S-1), platinum (cisplatin or oxaliplatin), taxane (paclitaxel or docetaxel), or epirubicin, irinotecan, or trastuzumab if HER2-positive, or ramucirumab; disease progression within 6 months after the last treatment, and Eastern Cooperative Oncology Group (ECOG) performance status (PS) 0 or 1.

Exclusion criteria included (1) malignancies other than adenocarcinoma of the stomach or GEJ (including hematological malignancies) within 2 years prior to randomization, (2) central nervous system metastases, (3) treatment for advanced/metastatic gastric cancer within 3 weeks prior to randomization, with cytotoxic chemotherapy, other targeted therapies (4 weeks for ramucirumab, mitomycin C, nitrosourea, or lomustine), immunotherapy or radiotherapy (local therapy for non-curative symptom relief was allowed until 2 weeks before randomization), (4) therapy with clinically significant systemic anticoagulant or antithrombotic agents within 7 days prior to randomization that could prevent blood clotting, (5) previous treatment with rivoceranib, and (6) known hypersensitivity to rivoceranib or components of the formulation.

The study was conducted in accordance with the relevant regulatory requirements, ethical principles consistent with the International Conference on Harmonisation of Technical Requirements for Registration of Pharmaceuticals for Human Use (ICH) Good Clinical Practice (GCP) guideline, and the Declaration of Helsinki. In addition, investigational site personnel complied with regional or country standard operating procedures and local regulatory and ethical requirements. All patients provided written informed consent prior to enrollment.

### Randomization and masking

Eligible patients were randomized in a 2:1 ratio to rivoceranib plus BSC or placebo plus BSC. Randomization was stratified by geographic region (Asia vs. North America/Europe), measurable disease (measurable vs. non-measurable), prior ramucirumab treatment (yes vs. no), and treatment line (3rd vs. ≥4th). Randomization was balanced with randomly permuted blocks and implemented with an interactive web-response system, which assigned a unique code that dictated the treatment assignment and matching study drug kit for each patient. Thus, treatment assignments were masked from patients, study personnel, and the funder.

### Procedures

Patients received oral rivoceranib 700 mg once daily (qd) or matching placebo approximately 1 h after breakfast plus BSC. The patients continued study treatment until disease progression or intolerable toxicity, withdrawal of consent, or death. Patients were allowed to continue study treatment after radiological disease progression at the discretion of the investigator. If treatment-related toxicity was detected, two rivoceranib dose reductions to 600 mg qd then 400 mg qd were allowed during the entire study period. Patients were evaluated at regular site visits, every 2 weeks. Tumor response and progression were assessed every 8 weeks at a local imaging facility. Evaluation of disease was performed at baseline and throughout the study both by the investigator and by blinded independent central review (BICR) after study completion. Patients’ best tumor response and time of progression were assessed according to RECIST version 1.1. A post-treatment follow-up visit was made at 4 weeks after the end of treatment and patients were then followed-up for survival at 8-week intervals until death or study closure. Safety was evaluated based on National Cancer Institute (NCI) Common Terminology Criteria for Adverse Events (CTCAE) v4.03 [18].

### Outcomes

The primary endpoint was OS (defined as the time from randomization to death) in the intention-to-treat (ITT) population. Secondary efficacy endpoints included PFS (defined as the time from randomization to either progression by central review or death), the objective response rate (ORR, defined as the proportion of patients with a best overall response of complete response [CR] or partial response [PR]) and the disease control rate (DCR, defined as the proportion of patients with a best overall response of CR, or PR, or stable disease [SD]). The ORR was reported for patients with measurable disease at baseline. Safety and tolerability were secondary endpoints.

### Statistical analysis

Assuming median OS of 6.53 months and 4.7 months for rivoceranib and placebo, respectively, corresponding to a hazard ratio [HR] of 0.72, a total of 325 events (413 patients) was needed to provide approximately 80% power with a two-sided significance level of alpha = 0.05 for OS. Assuming a 10% drop out rate, a total of 459 patients (306 for rivoceranib and 153 for placebo) were planned to be randomized to the two treatment arms. The ITT population, consisting of all randomly assigned patients, was used for the primary efficacy analyses.

To control the family-wise error rate (FWER) on testing multiple hypotheses of interest, a fixed sequence closed testing procedure was used, in which each hypothesis was sequentially tested with a two-sided 5% level only if the higher-level null hypothesis was rejected. For this fixed sequential testing, the primary and key secondary efficacy endpoints were tested using the ITT set: (1) OS was tested with a two-sided 5% level; (2) If OS was significant, PFS (based on BICR) was tested with a two-sided 5% level; (3) if OS and PFS were significant, ORR (based on BICR of patients with measurable lesions) was tested with a two-sided 5% level. If a prior test was not statistically significant, subsequent analyses were descriptive rather than confirmatory.

Comparison of OS and PFS between the two treatment arms was performed with a log-rank test stratified by the randomization stratification variables (geographic region, measurable disease, prior ramucirumab treatment, and treatment line). The HRs were estimated using a Cox proportional hazards regression model fitted with treatment arm as a factor and the randomization stratification variables as covariates. ORR and DCR were compared using the Cochran–Mantel–Haenszel test stratified by the randomization stratification factors.

### Role of the funding source

Study drug and funding was provided by the sponsor. The sponsor collaborated with investigators to design the protocol, collect, analyze, and interpret data, and to prepare the study manuscript.

## Results

### Patients

Between March 14, 2017 and October 31, 2018, 460 patients were randomized, 308 to rivoceranib plus BSC and 152 to placebo plus BSC across 95 sites in 12 countries (Online Resource Fig. S1). Of the randomized patients, only one patient from both the rivoceranib and placebo arms failed to receive study drug. Patient characteristics were similar between the two treatment arms, including the four stratification factors (Table 1). The median age in the ITT population was 60 years and most patients (76.7%) were male. Approximately two thirds of patients were Asian (67.8%) and one third were White (31.7%). The stomach was the primary tumor site in 87.6% of patients and 43.9% of patients had liver metastases. Most patients (59.8%) received study treatment as 3rd-line therapy in this study. The most common anticancer therapies prior to study entry were fluoropyrimidines (intravenous 5-FU, capecitabine, and S-1), platinum-containing compounds (oxaliplatin and cisplatin), taxanes (paclitaxel and docetaxel), irinotecan, ramucirumab, trastuzumab, immunotherapies (nivolumab and pembrolizumab), and epirubicin (Online Resource Tables S1, S2); 32.4% had received prior ramucirumab.

**Table 1:** Patient baseline characteristics

	Rivoceranib + BSC (*n* = 308)	Placebo + BSC (*n* = 152)
Age, years	60.0 (21.0–91.0)	61.0 (27.0–82.0)
Male	241 (78.2%)	112 (73.7%)
Geographic region		
Asia Pacific	207 (67.2%)	103 (67.8%)
North America/Europe	101 (32.8%)	49 (32.2%)
Race		
Asian	207 (67.2%)	105 (69.1%)
White	100 (32.5%)	46 (30.3%)
Other	1 (0.3%)	1 (0.7%)
Disease measurability		
Measurable	262 (85.1%)	130 (85.5%)
Non-measurable	46 (14.9%)	22 (14.5%)
Treatment therapy line		
3rd	186 (60.4%)	89 (58.6%)
≥4th	122 (39.6%)	63 (41.4%)
Prior ramucirumab treatment	102 (33.1%)	49 (32.2%)
ECOG PS		
0	82 (26.6%)	35 (23.0%)
1	226 (73.4%)	117 (77.0%)
Primary tumor site		
Gastric	274 (89.0%)	129 (84.9%)
Gastroesophageal junction	34 (11.0%)	23 (15.1%)
Previous gastrectomy	148 (48.1%)	78 (51.3%)
No. of organs with metastases		
<2	112 (36.4%)	59 (38.8%)
≥2	196 (63.6%)	93 (61.2%)
Liver metastases	134 (43.5%)	68 (44.7%)

Data are number of patients (%) or median (range)

*BSC* best supportive care; *ECOG PS* Eastern Cooperative Oncology Group performance status

### Efficacy

At the database lock for the primary analysis (30 May 2019), 83.1% of rivoceranib patients and 81.6% of placebo patients had discontinued study participation; 12.7% of rivoceranib patients were in follow-up and 4.2% remained on treatment versus 15.1% of placebo patients in follow-up and 3.3% on treatment. The main reasons for treatment discontinuation were disease progression (67.2% rivoceranib vs. 79.6% placebo), adverse events (AEs; 15.9% vs. 5.9%), and withdrawal of consent (6.8% vs. 6.6%).

**Fig. 1:**
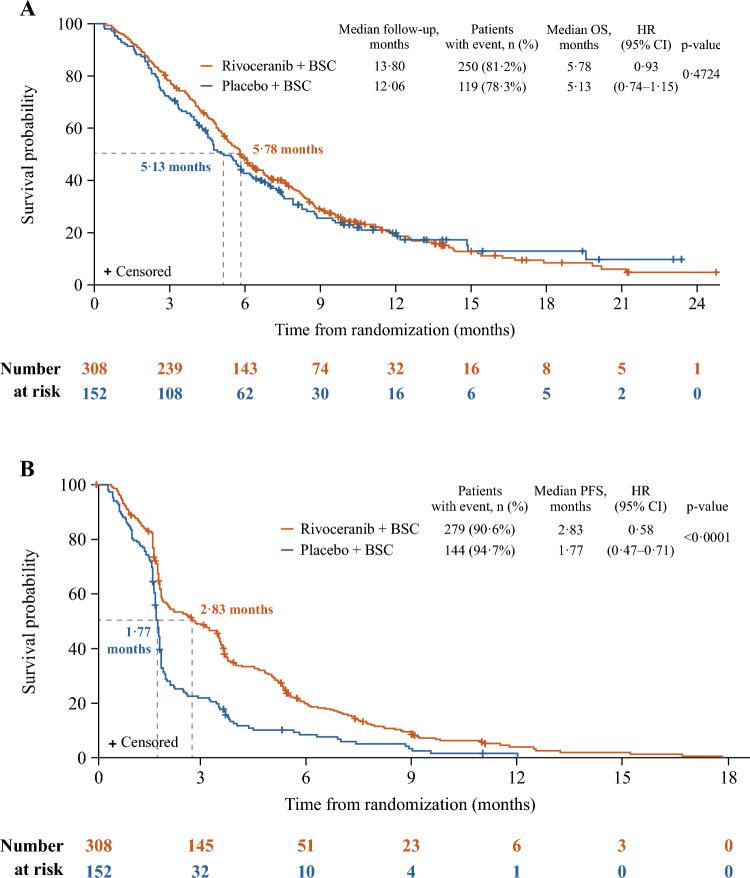
Kaplan–Meier estimates of **A** overall survival and **B** progression-free survival in the ITT population. HR = hazard ratio. ITT = intention-to-treat. OS = overall survival. PFS = progression-free survival

The primary study analysis was performed after a total of 369 OS events were recorded (30 May 2019). The median follow-up time in all patients was 13.80 months for rivoceranib and 12.06 months for placebo. In the ITT population, median OS was 5.78 months for rivoceranib and 5.13 months for placebo (HR 0.93, 95% CI 0.74–1.15; *p* = 0.4724; Fig. 1A), with a 7% reduction in the risk of death. As the primary endpoint was not met, all subsequent analyses were descriptive rather than confirmatory, as discussed above. Median PFS by BICR was 2.83 months for rivoceranib and 1.77 months for placebo (HR 0.58, 95% CI 0.47–0.71; *p* < 0.0001; Fig. 1B). BICR of patients in the ITT population indicated two patients (0.6%) had a CR, 107 (34.7%) had progressive disease (PD), and 19 (6.2%) had non-CR/non-PD in the rivoceranib arm compared with one patient (0.7%) with a CR, 92 (60.5%), with PD, and 12 (7.9%) with non-CR/non-PD in the placebo arm (Table 2). In the ITT population, 23 (7.5%) and six patients (3.9%) were not evaluable, and data were missing for 35 (11.4%) and 22 patients (14.5%) in the rivoceranib and placebo groups, respectively. In the measurable disease population, 16 patients (6.1%) in the rivoceranib arm had a PR versus no patients (0%) in the placebo arm (Table 2). Rivoceranib improved the ORR versus placebo in patients with measurable disease at baseline (6.9% vs. 0%, respectively; *p* = 0.0020) and the DCR versus placebo in the ITT population (40.3% vs. 13.2%, respectively; *p* < 0.0001) (Table 2). The percentage changes in the sum of the target lesions from baseline over time in patients with measurable lesions (*n* = 392) are shown in Online Resource Fig. S2.

**Table 2:** Response and disease control rate in patients with measurable disease at baseline

	Patients with measurable disease	
	Rivoceranib + BSC (*n* = 262)	Placebo + BSC (*n* = 130)
Objective response rate	18 (6.9%)	0 (0.0%)
(95% CI)	(4.12–10.64)	(0.00–2.80)
*p* value	0.002	
Best overall response		
Complete response	2 (0.8%)	0 (0.0%)
Partial response	16 (6.1%)	0 (0.0%)
Stable disease	93 (35.5%)	17 (13.1%)
Progressive disease	98 (37.4%)	81 (62.3%)
Not evaluable	18 (6.9%)	6 (4.6%)
Missing	30 (11.5%)	20 (15.4%)
Disease control rate	111 (42.4%)	17 (13.1%)
(95% CI)	(36.38–48.35)	(7.28–18.87)
*p* value	< 0.0001	

	ITT population	
	Rivoceranib + BSC (*n* = 308)	Placebo + BSC (*n* = 152)
Objective response rate	20 (6.5%)	2 (1.3%)
(95% CI)	(3.74–9.25)	(0.16–4.67)
*p* value	0.0119	
Best overall response		
Complete response	2 (0.6%)	1 (0.7%)
Partial response	18 (5.8%)	1 (0.7%)
Stable disease	104 (33.8%)	18 (11.8%)
Non-CR/non-PD	19 (6.2%)	12 (7.9%)
Progressive disease	107 (34.7%)	92 (60.5%)
Not evaluable	23 (7.5%)	6 (3.9%)
Missing	35 (11.4%)	22 (14.5%)

Data are number of patients (%)

*BSC* best supportive care; *ITT* intention-to-treat; *CR* complete response; *PD* progressive disease

**Fig. 2:**
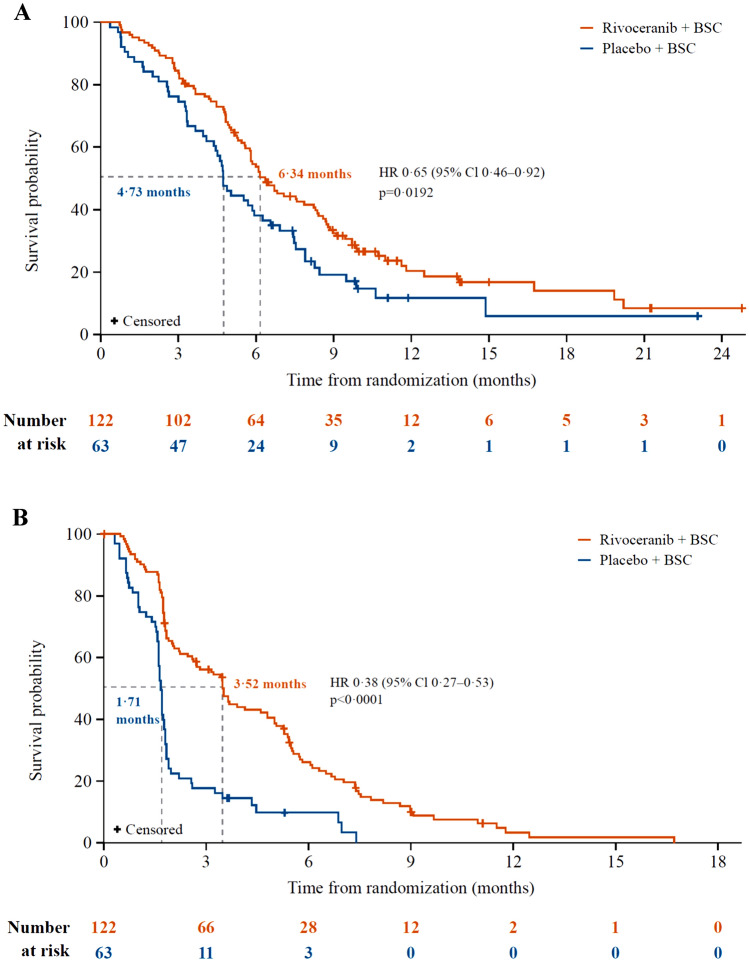
Kaplan–Meier estimates of **A** overall survival and **B** progression-free survival in patients receiving rivoceranib as ≥4th-line treatment. BSC = best supportive care. HR = hazard ratio. OS = overall survival. PFS = progression-free survival

Analysis of the prespecified stratification factor for patients receiving treatment as ≥4th-line therapy (rivoceranib *n* = 122, placebo *n* = 63) revealed that both OS (median 6.34 vs. 4.73 months, respectively, HR 0.65, 95% CI 0.46–092; *p* = 0.0192) and PFS by BICR (median 3.52 vs. 1.71 months, respectively, HR 0.38, 95% CI 0.27–0.53; *p* < 0.0001) were improved with rivoceranib versus placebo (Fig. 2). However, for patients receiving treatment in the 3rd line setting, OS favored the placebo arm (5.32 months vs. 5.62 months, HR 1.15, 95% CI 0.86, 1.53). In addition, the ORR (9.0% vs. 0%; *p* = 0.0181) and DCR (50.5% vs. 10.7%; *p* < 0.0001) were also improved with rivoceranib versus placebo.

**Fig. 3:**
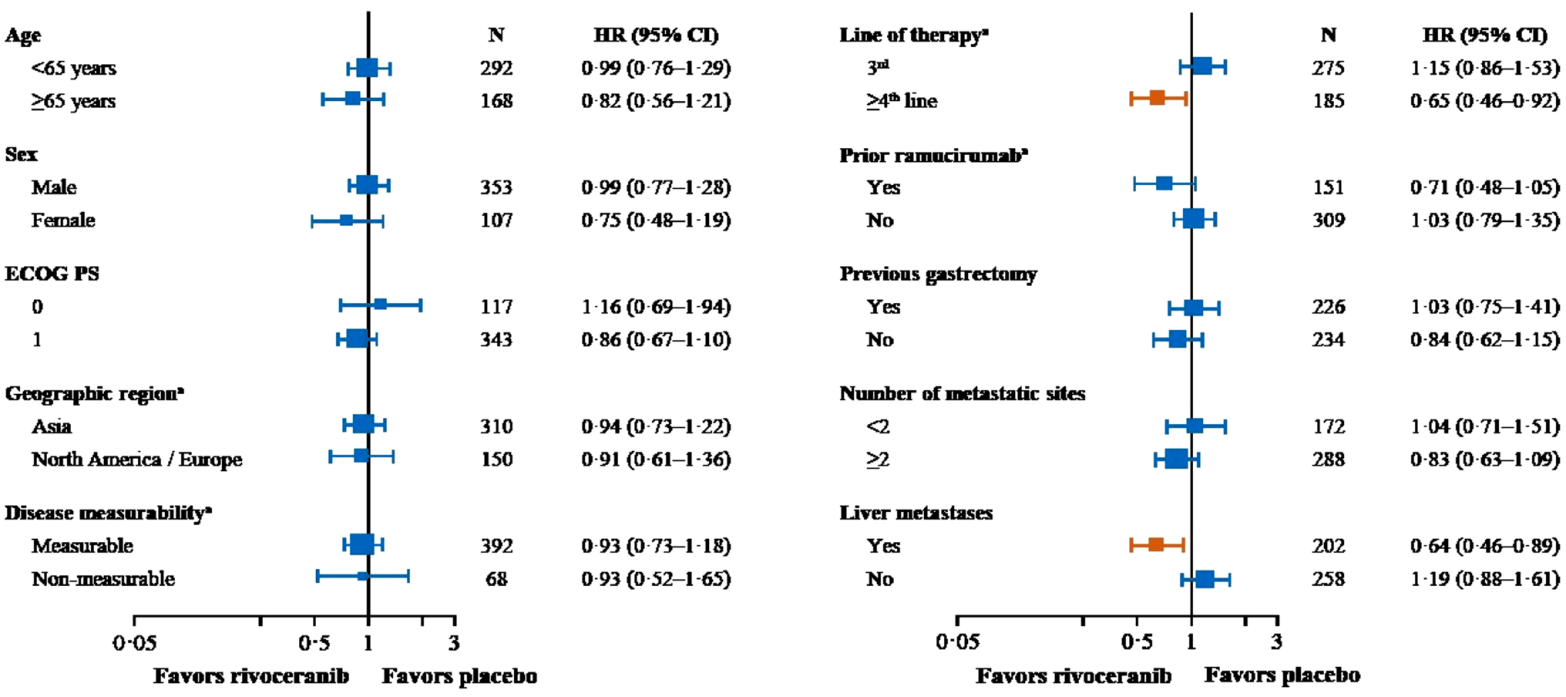
Forest plot of hazard ratios for overall survival by patient subgroups. ECOG PS = Eastern Cooperative Oncology Group performance status. HR = hazard ratio. ^a^ Stratification factors

In subgroup analyses, OS was relatively longer with rivoceranib versus placebo in the following patient subgroups: age ≥ 65 years versus <65 years (HR 0.82, 95% CI 0.56–1.21 vs. HR 0.99, 95% CI 0.76–1.29); ECOG PS 1 versus 0 (HR 0.86, 95% CI, 0.67–1.10 vs. HR 1.16, 95% CI 0.69–1.94); ≥2 versus <2 metastatic sites (HR 0.83, 95% CI 0.63–1.09 vs. HR 1.04, 95% CI 0.71–1.51); and presence versus absence of liver metastases (HR 0.64, 95% CI 0.46–0.89 vs. HR 1.19, 95% CI 0.88–1.61) (Fig. 3). In patients who received prior ramucirumab, median OS was 5.98 versus 4.73 months in the rivoceranib versus placebo arms (HR 0.71, 95% CI 0.48–1.05) compared with 5.78 versus 5.62 months (HR 1.03, 95% CI 0.79–1.35) in patients who did not receive prior ramucirumab.

For the rivoceranib arm versus placebo arm, respectively, in the ITT population, median OS was 5.39 months versus 4.14 months in White patients (HR 0.89, 95% CI 0.59–1.34) and 6.05 months versus 5.82 months in Asian patients (HR 0.94, 95% CI 0.73–1.22). Median PFS by BICR was 2.14 versus 1.81 months in White patients and 3.25 versus 1.74 months in Asian patients. The ORR in patients with measurable disease was 5.6% versus 0% in White patients and 9.5% versus 0% in Asian patients. The DCR in patients with measurable disease was 44.4% versus 0% in White patients and 52.7% versus 12.8% in Asian patients.

At the time of the OS analysis, post-study anticancer treatment data were available for 372 patients (80.9%). Post-study anticancer treatment was documented for 102 patients (33.1%) in the rivoceranib arm and 47 patients (30.9%) in the placebo arm. Taxanes were the most common post-study anticancer drug class and were received by 32 patients (10.4%) and 18 patients (11.8%) in the rivoceranib arm and placebo arm, respectively. Of the agents approved during the course of the study (eg, nivolumab, pembrolizumab, trastuzumab, and Tas 102), nivolumab was the most commonly used as post-study treatment (23 patients [7.5%] in the rivoceranib arm and 9 patients [5.9%] in the placebo arm). Among patients who received study treatment as ≥4th-line therapy, post-study anticancer treatment was documented for 44 patients (36.1%) in the rivoceranib arm and 20 patients (31.7%) in the placebo arm. In this subset, for the rivoceranib and placebo arms, respectively, the most common post-study treatments were nivolumab in 11 patients (9.0%) and 3 patients (4.8%), taxanes in 9 patients (7.4%) and 7 patients (11.1%), and irinotecan in 8 patients (6.5%) and 4 patients (6.3%).

### Safety

The safety population included 458 patients who received at least one dose of rivoceranib or placebo (rivoceranib *n* = 307, placebo *n* = 151) (Online Resource Fig. S1). Median duration of treatment was 1.91 months in the rivoceranib arm versus 1.78 months in the placebo arm. Patients assigned to the rivoceranib treatment arm received a mean (±SD) daily dose of 593.1 (±22.4) mg (84.7% of the protocol specified daily dose of 700 mg) while placebo patients received a mean (±SD) daily dose of 678.8 (±67.9) mg (97.0% of the protocol specified daily dose of 700 mg). Dose reductions of rivoceranib were required in 49.5% of patients and dose interruptions were required in 82.7% compared with 6.6% and 57.6%, respectively, for placebo. AEs were the most common reasons in rivoceranib and placebo patients for dose reduction (42.0% and 5.3%, respectively) and interruption (66.1% and 35.1%, respectively).

AEs of all grades were recorded in 306 (99.7%) and 144 (95.4%) patients receiving rivoceranib and placebo, respectively, while serious AEs were recorded in 146 (47.6%) and 66 (43.7%) patients receiving rivoceranib and placebo, respectively. Deaths attributed to AEs of any cause were observed in 21 (6.8%) and 11 (7.3%) patients receiving rivoceranib and placebo, respectively. Treatment-related AEs of all grades were recorded in 266 (86.6%) and 86 (57.0%) patients receiving rivoceranib and placebo, respectively.

The most common all-grade treatment-emergent AEs of any cause in the rivoceranib arm were decreased appetite (42.3%), hypertension (34.2%), proteinuria (29.3%), and diarrhea (29.3%). The most common grade ≥ 3 AEs in the rivoceranib arm were hypertension (17.9%), anemia (10.4%), aspartate aminotransferase increased (9.4%), asthenia (8.5%), and proteinuria (7.5%) (Table 3). Notably, the rates of grade ≥ 3 anemia (10.4% vs 15.9%) and asthenia (8.5% vs 9.9%) were lower in the rivoceranib arm compared with the placebo arm. Treatment-related AEs resulting in death occurred in 8 (2.6%) patients in the rivoceranib arm and 2 (1.3%) patients in the placebo arm. These events in the rivoceranib arm consisted of gastric hemorrhage in 2 (0.7%) patients and one patient (0.3%) each experienced intestinal infarction, acute myocardial infarction, pneumonia, subdural hemorrhage, cachexia, and acute kidney injury. In the placebo arm these events consisted of sudden death and hepatitis in one patient each (0.7%). Three (2.5%) patients in the ≥4th line subgroup had a treatment-related AE resulting in death, all in the rivoceranib arm, which consisted of intestinal infarction, subdural hemorrhage, and an acute kidney injury (1 patient [0.8%] each).

**Table 3:** Treatment-emergent adverse events occurring in ≥20% of patients receiving rivoceranib

Adverse event	Rivoceranib + BSC (*n* = 307)		Placebo + BSC (*n* = 151)	
	All grades	Grade ≥ 3	All grades	Grade ≥ 3
Any adverse event	306 (99.7%)	234 (76.2%)	144 (95.4%)	85 (56.3%)
Decreased appetite	130 (42.3%)	22 (7.2%)	48 (31.8%)	7 (4.6%)
Hypertension	105 (34.2%)	55 (17.9%)	5 (3.3%)	0 (0.0%)
Proteinuria	90 (29.3%)	23 (7.5%)	11 (7.3%)	0 (0.0%)
Diarrhea	90 (29.3%)	10 (3.3%)	20 (13.2%)	0 (0.0%)
Asthenia	87 (28.3%)	26 (8.5%)	35 (23.2%)	15 (9.9%)
Abdominal pain	85 (27.7%)	22 (7.2%)	31 (20.5%)	7 (4.6%)
Palmar–plantar erythrodysesthesia syndrome	81 (26.4%)	9 (2.9%)	6 (4.0%)	0 (0.0%)
Fatigue	75 (24.4%)	17 (5.5%)	16 (10.6%)	5 (3.3%)
Nausea	71 (23.1%)	5 (1.6%)	34 (22.5%)	0 (0.0%)
Aspartate aminotransferase increased	71 (23.1%)	29 (9.4%)	11 (7.3%)	3 (2.0%)
Stomatitis	69 (22.5%)	11 (3.6%)	5 (3.3%)	0 (0.0%)
Weight decreased	68 (22.1%)	6 (2.0%)	12 (7.9%)	0 (0.0%)
Alanine aminotransferase increased	63 (20.5%)	17 (5.5%)	9 (6.0%)	2 (1.3%)
Anemia	64 (20.8%)	32 (10.4%)	41 (27.2%)	24 (15.9%)

Events are listed if they occurred in ≥20% of patients receiving rivoceranib (all grades)

Data are number of patients (%)

*BSC* best supportive care

## Discussion

Over the past 8 years, a number of monotherapy trials investigating new agents for the 3rd-line treatment of advanced/metastatic gastric cancer have been completed (Table 4). Many of these studies were global, such as the phase 3 study of trifluridine/tipiracil (TAGS) [7], the phase 2 study of pembrolizumab (KEYNOTE-059) [8], and the phase 3 study of ramucirumab (REGARD) [19], while others were regional, such as the nivolumab ATTRACTION-2 study [9], or conducted in a single country, such as the apatinib phase 3 study conducted in China [14]. While cross-study comparisons warrant caution due to differences in study populations and standards of care in each country, there has been a high degree of similarity in median OS and median PFS in the placebo arms among recent studies in the 3rd-line advanced gastric cancer setting. The median OS in the placebo arms averaged approximately 4 months (range 3.6–4.7) while the median PFS averaged approximately 1.6 months (range 1.3–1.8) [7, 9, 14, 19].

**Table 4:** Clinical trials in late-line gastric cancer

	Rivoceranib vs. placebo (ANGEL)	Trifluridine/tipiracil vs. placebo (TAGS) [7]	Nivolumab vs. placebo (ATTRACTION-2) [9]	Pembrolizumab single arm (KEYNOTE-059) [8]	Rivoceranib vs. placebo [14]	Ramucirumab vs. placebo (REGARD) [19]
Date of study	Mar 2017 to Sep 2020	Feb 2016 to Jan 2018	Nov 2014 to Feb 2016	Mar 2015 to May 2016	Jan 2011 to Nov 2012	Oct 2009 to Jan 2012
Regions	North America, Europe, Asia Pacific	North America, Europe, Asia Pacific, Middle East	Asia Pacific	North America, Europe, Asia Pacific, Middle East, Australia	Asia Pacific (China only)	North, Central, South America; Europe, Asia, Australia, Africa
Line of therapy	≥3rd	≥3rd	≥3rd	≥3rd	≥3rd	≥2nd
Sample size (randomization ratio)	460 (2:1)	507 (2:1)	493 (2:1)	259 (single arm)	273 (2:1)	355 (2:1)
Median OS active vs. placebo (difference)	5.78 vs. 5.13 (0.65)	5.7 vs. 3.6 (2.1)	5.26 vs. 4.14 (1.12)	5.6 vs. NA (NA)	6.5 vs. 4.7 (1.8)	5.2 vs. 3.8 (1.4)
Median PFS active vs. placebo (difference)	2.83 vs. 1.77 (1.06)	2.0 vs. 1.8 (0.2)	1.61 vs. 1.45 (0.16)	2.0 vs. NA (NA)	2.6 vs. 1.8 (0.8)	2.1 vs. 1.3 (0.8)
ORR active vs. placebo	6.5% vs. 1.3%	4% vs. 2%	11.2% vs. 0%	11.6% vs. NA	2.8% vs. 0.0%	3% vs. 3%
DCR active vs. placebo	40.3% vs. 13.2%	44% vs. 14%	40% vs. 25%	27.0% vs. NA	42.1% vs. 31.8%	49% vs. 23%

Data are number of patients, months, or %

*OS* overall survival; *PFS* progression-free survival; *ORR* objective response rate; *DCR* disease control rate; *NA* not applicable

The ANGEL study was designed and powered based on a survival outcome expected to be similar to that seen in the Chinese phase 3 study of apatinib, which showed a 1.8-month improvement in OS with rivoceranib versus placebo (6.5 vs. 4.7 months) [14]. Ultimately, the ANGEL study did not demonstrate a statistically significant improvement in the primary endpoint, OS in the ITT population, with a numerical improvement in median OS of 0.7 months versus placebo and a 7% reduction in the risk of death. The improvements in secondary efficacy endpoints, specifically the prolongation in median PFS of 1.0 month versus placebo, the notable ORR of 6.5% with rivoceranib compared with 1.3% for placebo, and the DCR of 40.3% versus 13.2%, respectively, were not reflected in the ITT OS results.

While improvement of OS remains the gold standard for oncology studies and the ultimate goal for new therapies, it remains an endpoint that can be easily confounded or diluted, principally by post-study anticancer therapies. Whether post-study anticancer therapies affected our results is unclear. At the time of the primary OS analysis, post-study anticancer therapy data were unavailable for 19.1% of patients. In addition, among patients receiving study treatment in the ≤3rd line, additional lines of therapy after 4th line were not captured.

Apart from the Chinese phase 3 trial of apatinib, the median OS of 5.8 months with rivoceranib in this study is the longest among those reported in global pivotal studies of ≥3rd-line therapy, as is the median PFS of 2.8 months. Possibly, the reason why this study did not meet the primary endpoint was the unparalleled median OS of 5.1 months observed in the placebo arm, which is the longest reported for placebo in studies of advanced/metastatic gastric cancer. Prior to the ANGEL study, the longest reported median OS with placebo in this population was 4.7 months in Chinese patients [14] and 3.6 months in the global TAGS study [7]. Similarly, the nivolumab ATTRACTION-2 study, conducted in Japan, South Korea, and Taiwan, reported a median OS of 4.1 months with placebo [9]. At the commencement of this study, there were no approved therapies in the 3rd-line setting (with the exception of apatinib in China). However, by the time of completion, three additional agents had been approved: nivolumab in Korea, Japan, and Taiwan; pembrolizumab in the USA for PDL1-positive patients; and trifluridine/tipiracil in the USA. Thus, the reason for the longer median OS with placebo in the ANGEL study compared with other studies may be because better post-study anticancer therapies were used in the ANGEL study, indicating that failure to meet the primary endpoint (i.e., OS) in the ANGEL study may not necessarily translate as lack of efficacy of rivoceranib.

The clearest signal of OS benefit observed in this study was in patients who received rivoceranib as their ≥4th-line of treatment. The study was stratified by line of therapy (3rd versus ≥4th) in anticipation of different efficacy in patients with a greater number of prior therapies and more advanced disease. Patients receiving rivoceranib as ≥4th-line therapy comprised a substantial proportion of the study population (*n* = 185, 40.2%) and were equally balanced between the rivoceranib (*n* = 122, 39.6%) and placebo arms (*n* = 63, 41.5%). In this group, median OS was 6.3 months for rivoceranib and 4.7 months for placebo, with a significant 35% reduction in the risk of death, supporting the clinical efficacy of rivoceranib in this subgroup of patients with advanced/metastatic gastric cancer.

Among the subgroup of patients who received prior ramucirumab, a 29% relative reduction in risk of death was observed with rivoceranib compared to placebo, indicating that prior ramucirumab did not have a negative impact on the efficacy of rivoceranib. In fact, the HR for OS appeared to favor patients with prior ramucirumab treatment, supporting the use of rivoceranib after standard 2nd-line ramucirumab therapy with or without paclitaxel. This is similar to the results of other studies that have shown the continued benefit of antiangiogenic therapy in patients who have received antiangiogenic therapy in earlier treatment lines. For example, in the phase 2 RAINSTORM study in which patients with advanced gastric cancer received 1st-line therapy with S-1 plus oxaliplatin with or without ramucirumab followed by paclitaxel plus ramucirumab, the addition of ramucirumab to 1st-line therapy did not appear to have a significant impact on PFS of 2nd-line paclitaxel plus ramucirumab [20].

Hypertension, proteinuria, and palmar–plantar erythrodysesthesia (hand–foot) syndrome are among the most common AEs associated with non-selective TKIs that have anti-VEGF activity and, to a lesser extent, with the anti-VEGFR monoclonal antibody ramucirumab [19]. In the current study, hypertension and proteinuria were common grade ≥ 3 AEs irrespective of causality in the rivoceranib arm, occurring in 17.9% and 7.5% of patients, respectively. Palmar–plantar erythrodysesthesia syndrome grade ≥ 3 occurred in 2.9% of rivoceranib patients. In the phase 3 ramucirumab monotherapy REGARD study, hypertension and proteinuria grade ≥ 3 occurred in 8% and <1% of patients, respectively, in the ramucirumab arm [19]. Other common grade ≥ 3 adverse events in the ramucirumab arm were fatigue, abdominal pain, and anemia (all 6%) [19], each of which occurred at similar rates with rivoceranib in ANGEL (5.5%, 7.2%, and 10.4%, respectively). Based on the results of our study, the incidence of grade ≥ 3 AEs is similar between rivoceranib and ramucirumab.

A limitation of the study was the emergence of several new effective agents during the course of the trial. It is not clear what impact these post-study treatments may have had on the OS results.

In conclusion, OS was not significantly improved in the ITT population in this phase 3 study. However, secondary efficacy endpoints, including median PFS, ORR, and DCR, were improved in the ITT population. In addition, median OS and secondary efficacy endpoints, including median PFS, ORR, and DCR, were improved in patients receiving ≥4th-line therapy. The OS benefit observed in patients receiving rivoceranib in the ≥4th line warrants further study in a dedicated trial of this subgroup.

### Supplementary Information

Below is the link to the electronic supplementary material.

Supplementary file1 (DOCX 796 KB)
