# Supplementary material for: Rivoceranib, a VEGFR-2 inhibitor, monotherapy in previously treated patients with advanced or metastatic gastric or gastroesophageal junction cancer (ANGEL study): an international, randomized, placebo-controlled, phase 3 trial
Source: Gastric Cancer. 2024 Jan 28;27(2):375–86. doi: 10.1007/s10120-023-01455-5 (PMC10896803; doi:10.1007/s10120-023-01455-5)
Supplement: Supplementary file 1 — Supplementary file1 (DOCX 796 KB) [file 10120_2023_1455_MOESM1_ESM.docx]

# Appendix

***Table S1:* Anticancer therapies prior to study enrollment**

|  | Rivoceranib + BSC | Placebo + BSC |
| --- | --- | --- |
| **Agent** | (N=308) | (N=152) |
| **Fluoropyrimidines** |  |  |
| IV 5-FU | 167 (54.2%) | 84 (55.3%) |
| Capecitabine | 160 (51.9%) | 71 (46.7%) |
| S-1 | 65 (21.1%) | 37 (24.3%) |
| **Platinum-containing agents** |  |  |
| Oxaliplatin | 216 (70.1%) | 112 (73.7%) |
| Cisplatin | 107 (34.7%) | 52 (34.2%) |
| **Taxanes** |  |  |
| Paclitaxel | 160 (51.9%) | 84 (55.3%) |
| Docetaxel | 51 (16.6%) | 17 (11.2%) |
| **Immunotherapy** |  |  |
| Nivolumab | 27 (8.8%) | 12 (7.9%) |
| Pembrolizumab | 4 (1.3%) | 1 (0.7%) |
| **VEGF inhibition** |  |  |
| Ramucirumab | 100 (32.5%) | 49 (32.2%) |
| **Others** |  |  |
| Irinotecan | 143 (46.4%) | 80 (52.6%) |
| Trastuzumab | 44 (14.3%) | 24 (15.8%) |
| Epirubicin | 18 (5.8%) | 8 (5.3%) |
| Others | 146 (47.4%) | 59 (38.8%) |

Data are number of patients (%). BSC=best supportive care. IV=intravenous. FU=fluorouracil. VEGF=vascular endothelial growth factor.

***Table S2:* Anticancer therapies prior to study enrollment in the ≥4th -line subgroup**

|  | Rivoceranib + BSC | Placebo + BSC |
| --- | --- | --- |
| **Agent** | (N=122) | (N=63) |
| **Fluoropyrimidines** |  |  |
| IV 5-FU | 64 (52.5%) | 34 (54.0%) |
| Capecitabine | 60 (49.2%) | 28 (44.4%) |
| S-1 | 35 (28.7%) | 22 (34.9%) |
| **Platinum-containing agents** |  |  |
| Oxaliplatin | 79 (64.8%) | 45 (71.4%) |
| Cisplatin | 48 (39.3%) | 23 (36.5%) |
| **Taxanes** |  |  |
| Paclitaxel | 83 (68.0%) | 45 (71.4%) |
| Docetaxel | 19 (15.6%) | 9 (14.3%) |
| **Immunotherapy** |  |  |
| Nivolumab | 22 (18.0%) | 10 (15.9%) |
| Pembrolizumab | 4 (3.3%) | 1 (1.6%) |
| **VEGF inhibition** |  |  |
| Ramucirumab | 59 (48.4%) | 29 (46.0%) |
| **Others** |  |  |
| Irinotecan | 76 (62.3%) | 47 (74.6%) |
| Trastuzumab | 23 (18.9%) | 14 (22.2%) |
| Epirubicin | 9 (7.4%) | 2 (3.2%) |
| Others | 72 (59.0%) | 26 (41.3%) |

Data are number of patients (%). BSC=best supportive care. IV=intravenous. FU=fluorouracil. VEGF=vascular endothelial growth factor.

***Figure S1:* Patient disposition.**


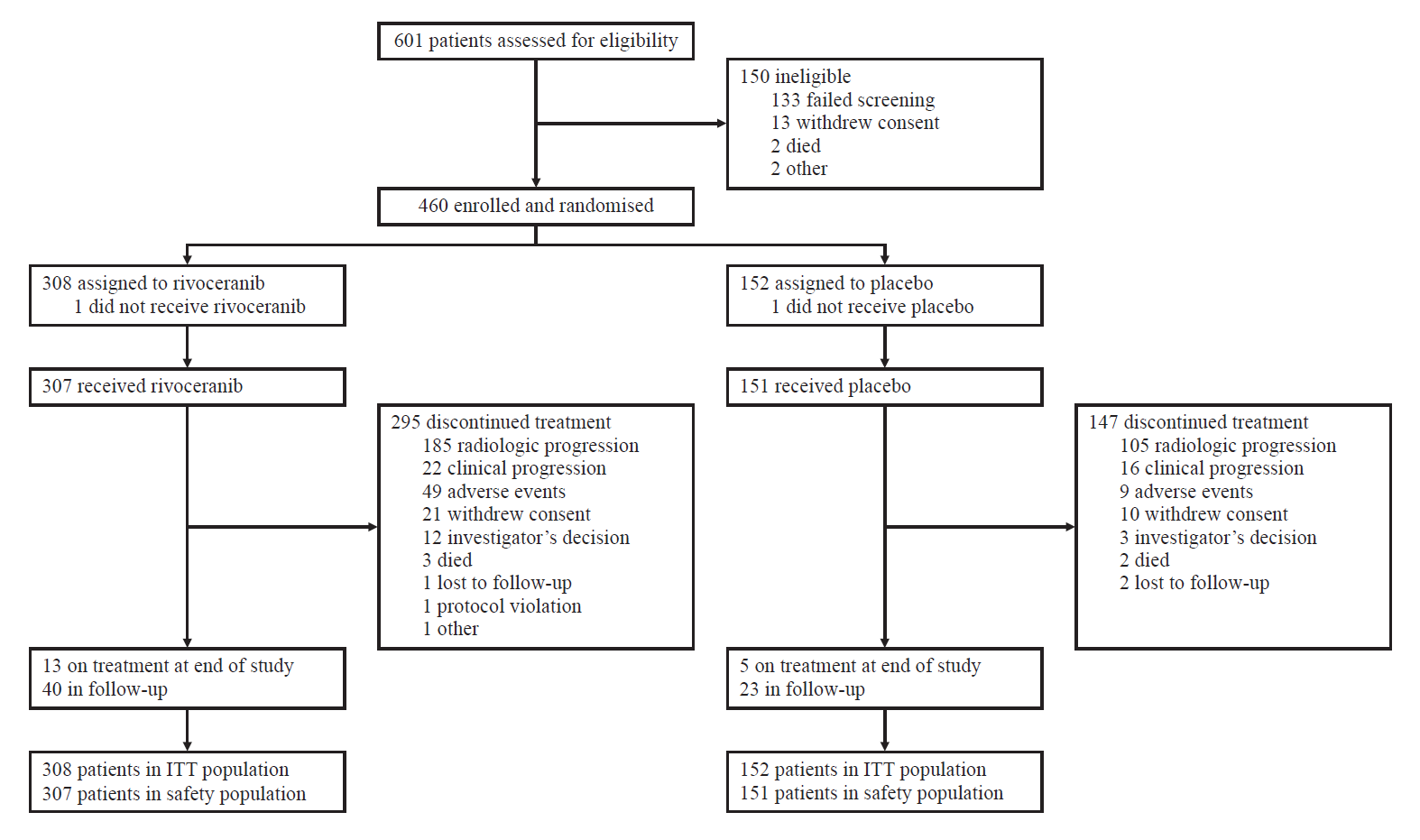


ITT=intention-to-treat

***Figure S2:* Percentage change in sum of target lesions from baseline over time (blinded independent central review)**


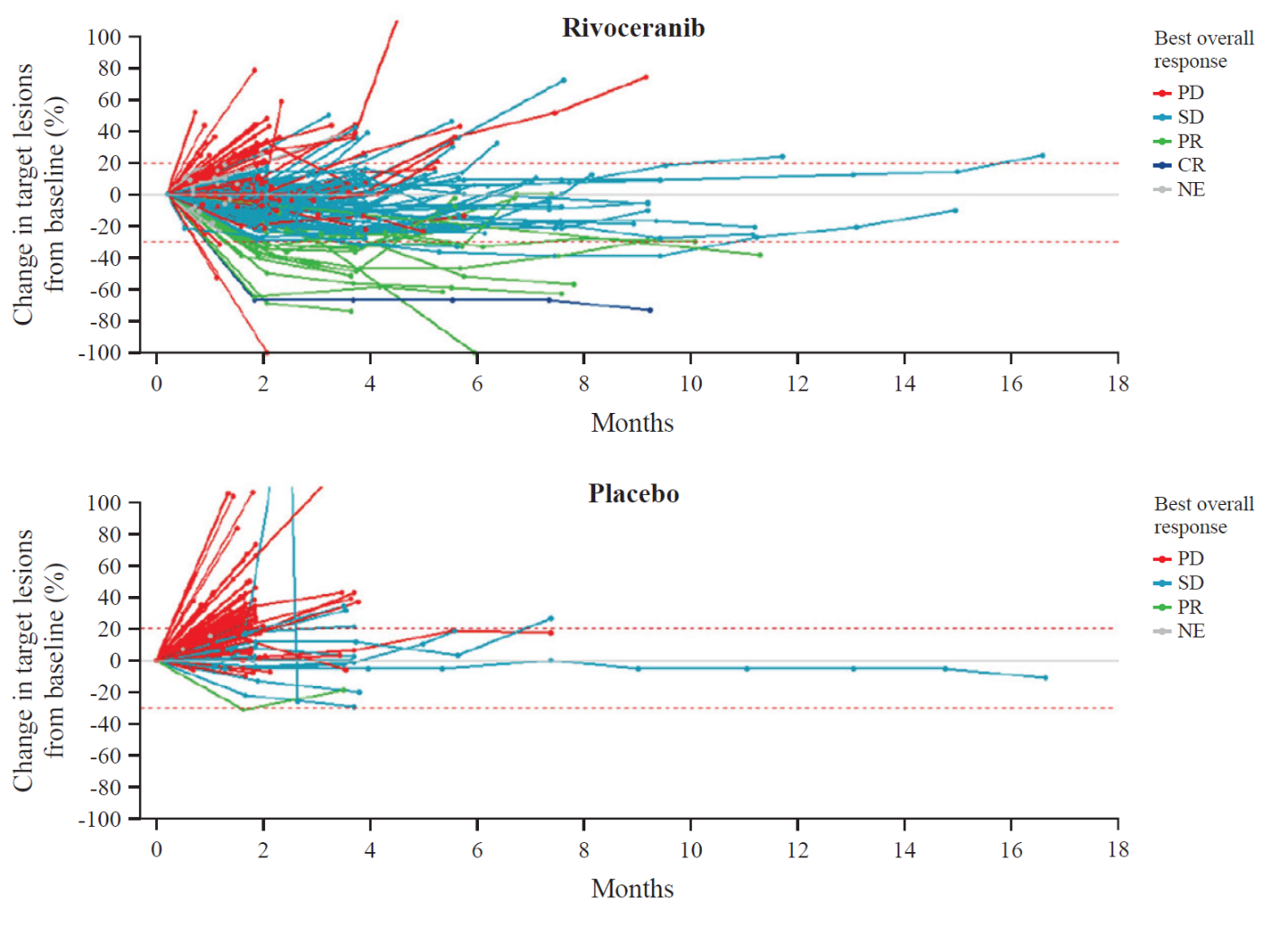


PD=progressive disease. SD=stable disease. PR=partial response. NE=not evaluable.
